# Higher serum phosphate within the normal range is associated with the development of calcified aortic valve disease

**DOI:** 10.3389/fcvm.2024.1450757

**Published:** 2024-09-26

**Authors:** Kyung An Kim, Hae-Ok Jung, Mi-Jeong Kim, So-Young Lee, Yuran Ahn, Mi-Hyang Jung, Woo-Baek Chung, Dong-Hyeon Lee, Ho-Joong Youn, Hyuk-Jae Chang

**Affiliations:** ^1^ Division of Cardiology, Department of Internal Medicine, Seoul St. Mary’s Hospital, The Catholic University of Korea, Seoul, Republic of Korea; ^2^ Division of Cardiology, Department of Internal Medicine, Incheon St. Mary’s Hospital, The Catholic University of Korea, Incheon, Republic of Korea; ^3^ Catholic Research Institute for Intractable Cardiovascular Disease, College of Medicine, The Catholic University of Korea, Seoul, Republic of Korea; ^4^ Division of Cardiology, Department of Internal Medicine, Uijeongbu St. Mary’s Hospital, The Catholic University of Korea, Uijeongbu, Republic of Korea; ^5^ Health Promotion Center, Seoul St. Mary’s Hospital, The Catholic University of Korea, Seoul, Republic of Korea; ^6^ Division of Cardiology, Yonsei Cardiovascular Center, Yonsei University Health System, Seoul, Republic of Korea

**Keywords:** calcified aortic valve disease, atherosclerosis, cardiac computed tomography, echocardiography, calcium-phosphate metabolism

## Abstract

**Background:**

Despite the essential role of ectopic osteogenic calcium-phosphate metabolism in the development of calcific aortic valve disease (CAVD), the implications of high serum phosphate levels in CAVD development are not fully understood.

**Methods:**

Asymptomatic individuals who underwent health screening using serial cardiac computed tomography (CT) and echocardiography were selected from a multicenter registry. CAVD was identified and quantified on CT images using the aortic valve calcification (AVC) score. The associations between initial serum phosphate levels and the presence of baseline CAVD, development of new CAVD, and the AVC score progression rate were investigated using multivariable regression models.

**Results:**

A total of 736 individuals were selected for analysis, and the median interscan duration was 36.4 months. On initial CT, 83 (13.7%) participants had baseline CAVD, while 52 (7.0%) individuals developed new CAVD during follow-up. Serum phosphate levels were not associated with a higher probability of baseline CAVD but were predictive of newly developed CAVD (odds ratio per 1 mg/dl, 1.05; 95% confidence interval, 1.01–1.10; *p* = 0.02). Higher phosphate levels were also associated with a faster AVC score progression in those with baseline CAVD (regression coefficient per 1 mg/dl, 15.55 Agatston units/year; 95% confidence interval, 6.02–25.07; *p* < 0.01), an association which remained significant when the analysis was extended to include newly developed CAVD.

**Conclusion:**

Even slight elevations in serum phosphate are associated with accelerated CAVD progression from an early stage. Further studies are needed to investigate whether the regulation of phosphate metabolism can slow the progression of CAVD to aortic stenosis.

## 1 Introduction

Calcific aortic valve disease (CAVD) is characterized by fibrotic thickening and calcification of the aortic valve (1). CAVD can progress to the point where the limitation of aortic valve opening becomes hemodynamically significant, which is referred to as aortic stenosis (AS)—a major cause of cardiovascular morbidity worldwide (2, 3). The detection of aortic valve calcification (AVC) on computed tomography (CT) is a sensitive and accurate method to quantify the calcific burden of CAVD and monitor disease progression (4, 5).

In the pathogenesis of CAVD, multiple pathways related to atherosclerosis and ectopic osteogenic calcium-phosphate metabolism are involved (6, 7). Although atherosclerotic risk factors are responsible for the initiation of CAVD, they are not associated with its progression (8, 9), and lipid lowering using statins did not succeed in preventing significant AS (10). Therefore, pathways related to osteogenesis and calcium-phosphate metabolism may be the key to developing pharmacologic interventions for CAVD (6, 11, 12). However, although elevated serum phosphate has been associated with the presence of CAVD, the implications of serum phosphate levels in CAVD progression are not fully understood (13, 14). Furthermore, the role of calcium-phosphate metabolism in CAVD may be of particular interest in the East Asian population, which has a lower burden of atherosclerosis compared with other ethnicities (3, 15, 16). Therefore, we sought to identify, among other factors, the relationship of serum phosphate levels with the progression of CAVD in ethnic Korean participants who were examined with cardiac CT scans as part of a general health examination.

## 2 Methods

### 2.1 Study design and population

The data used in this study were collected as part of the KOrea Initiatives on Coronary Artery calcification (KOICA) registry, which is a multicenter registry of individuals examined using cardiac CT scans during self-referred health examinations in six high-volume healthcare centers affiliated with tertiary hospitals in Korea. A total of 93,914 individuals were registered, and further details can be found in previous reports (16, 17). For this study, we selected the participants who were examined at Seoul St. Mary's Hospital, where same-day echocardiography was also part of the health examination. We limited our analysis to those with at least two CT scans during the study period (April 2009–July 2016) to identify factors associated with the progression of CAVD. Participants with bicuspid aortic valves were excluded, as they were thought to represent a heterogeneous population. Demographic factors, medical history, and current symptoms were self-reported using a detailed questionnaire. Medical records were retrospectively reviewed to ensure that the participants were free of cardiovascular symptoms at the initial or repeat CT scans which may have prompted the examination. Laboratory samples were drawn after a 12-hour fasting period and measured using an automatic analyzer (7,600–210; Hitachi Medical Corp., Tokyo, Japan). Lipid profiles were measured using a direct enzymatic method. The estimated glomerular filtration rate (eGFR) was calculated using the Chronic Kidney Disease Epidemiology Collaboration equation. The institutional review board of Seoul St. Mary's Hospital approved the study protocol (IRB KC23RISI0357) and waived the need for written informed consent because of the study's retrospective nature. The study protocol conforms to the ethical guidelines of the 1975 Declaration of Helsinki.

### 2.2 Data acquisition

Cardiac CT images were acquired using a 64-slice, dual-source CT scanner (SOMATOM Definition; Siemens, Forchheim, Germany). A non-contrast scan was first obtained using prospective triggering at 70% of the RR interval. The parameters used were tube voltage 120 kVp, gantry rotation time of 330 ms, and maximum tube current of 400 mA·s. Next, enhanced CT angiography scans were obtained using a retrospective electrocardiogram-gated protocol after the administration of 80–110 ml of iodinated contrast. CT images were transferred and reconstructed immediately after scanning using a computerized workstation (Advantage Windows Workstation 4.3; GE Healthcare, Milwaukee, WI, USA) with a slice thickness of 3 mm. Further details regarding the protocol for cardiac CT at our institution can be found in previous reports (18, 19).

CAVD was primarily assessed on CT images using a commercially available CT processing program (3mensio Structural Heart 10.0; Pie Medical Imaging, Maastricht, the Netherlands). As recommended in those undergoing evaluation for AS, the severity of CAVD was quantified on non-contrast axial images using the AVC score according to the Agatston method (4). Each calcified lesion from the base to the tip of the aortic valve leaflets was carefully selected, excluding calcifications found in the coronary arteries, aortic root, and left ventricular outflow tract. If necessary, the corresponding enhanced CT images were additionally referred to for the verification of anatomic structures. The AVC score was defined as the sum of the values for each calcified lesion and was assessed by two cardiologists (K.A.K. and S.-Y.L.) with 3 years of experience in cardiac imaging. A third cardiologist (H.-O.J.) with more than 20 years of cardiac imaging experience was consulted in cases of uncertainty.

The hemodynamic effects of CAVD were assessed using transthoracic echocardiography, which was performed by trained sonographers following standard guidelines (20). Images were obtained using commercially available ultrasound machines with 2.5- to 3.5-MHz transducers (GE Vivid E7 and Vivid E9, GE Healthcare, Chicago, IL, USA; Philips iE33, Amsterdam, the Netherlands). Because the majority of participants did not have findings suggestive of AS, continuous-wave Doppler measurements across the aortic valve were not routinely performed in these individuals.

### 2.3 Statistical analyses

The participants were classified into three groups according to the presence of CAVD on initial and subsequent CT scans: (1) no CAVD, defined as no AVC on any scan; (2) newly developed CAVD, defined as no AVC on the initial CT but with AVC score >0 on subsequent CT; (3) baseline CAVD. In the baseline characteristics, categorical data are presented as numbers and frequencies and compared using the *χ*2 test, and continuous variables are expressed as mean ± standard deviation and compared using one-way analysis of variance.

The relationship between serum phosphate and CAVD development was analyzed in a number of ways. First, the association between phosphate levels and both baseline and newly developed CAVD was analyzed using multivariable logistic regression models. Restricted cubic splines were also constructed to visualize the relationship between serum phosphate levels and other significant predictors with the probability of newly developed CAVD. Second, the association between phosphate levels and CAVD progression was analyzed using multivariable linear regression on the annualized AVC score progression rate in those with baseline CAVD. Finally, the association between CAVD progression and serum phosphate levels was analyzed in the entire group including both baseline CAVD and newly developed CAVD using a multivariable linear mixed-effects model with individual intercepts and the repeated effect estimated for time. Subgroup analysis was also performed using the multivariable linear mixed-effects model to confirm the effect of phosphate levels on CAVD progression in different populations. The relationship between serum phosphate levels and echocardiographic parameters was additionally explored using multivariable linear and logistic regression models.

Statistical analyses were performed using R version 4.3.2 (R Foundation for Statistical Computing, Vienna, Austria), and a two-sided *p* value <0.05 was considered statistically significant. Further details of the statistical methods used in this study can be found in the Supplementary Material.

## 3 Results

### 3.1 Baseline characteristics

**Figure 1:**
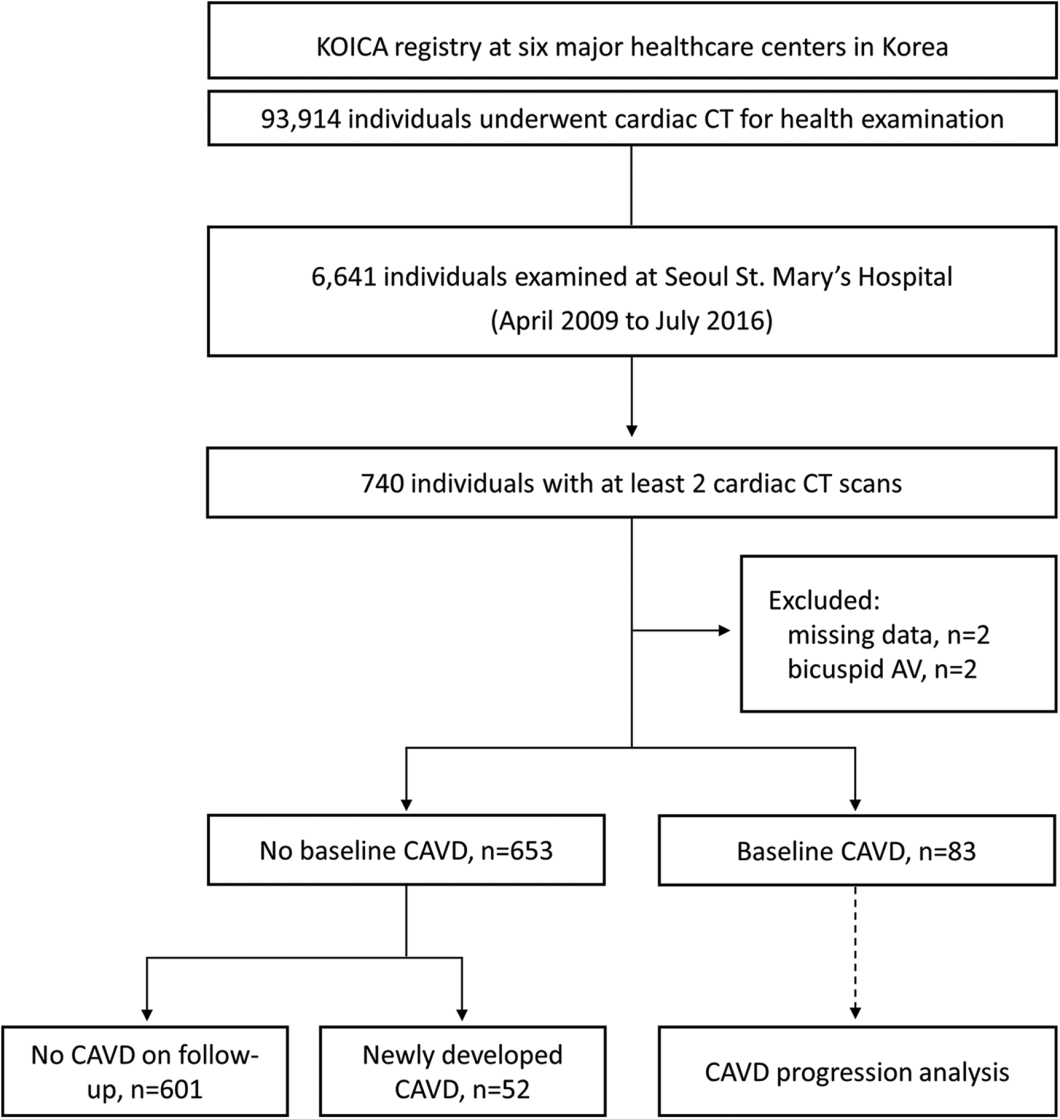
Selection process of the study population. KOICA, Korea initiatives on coronary artery calcification; CT, computed tomography; AV, aortic valve; CAVD, calcified aortic valve disease.

**Figure 2:**
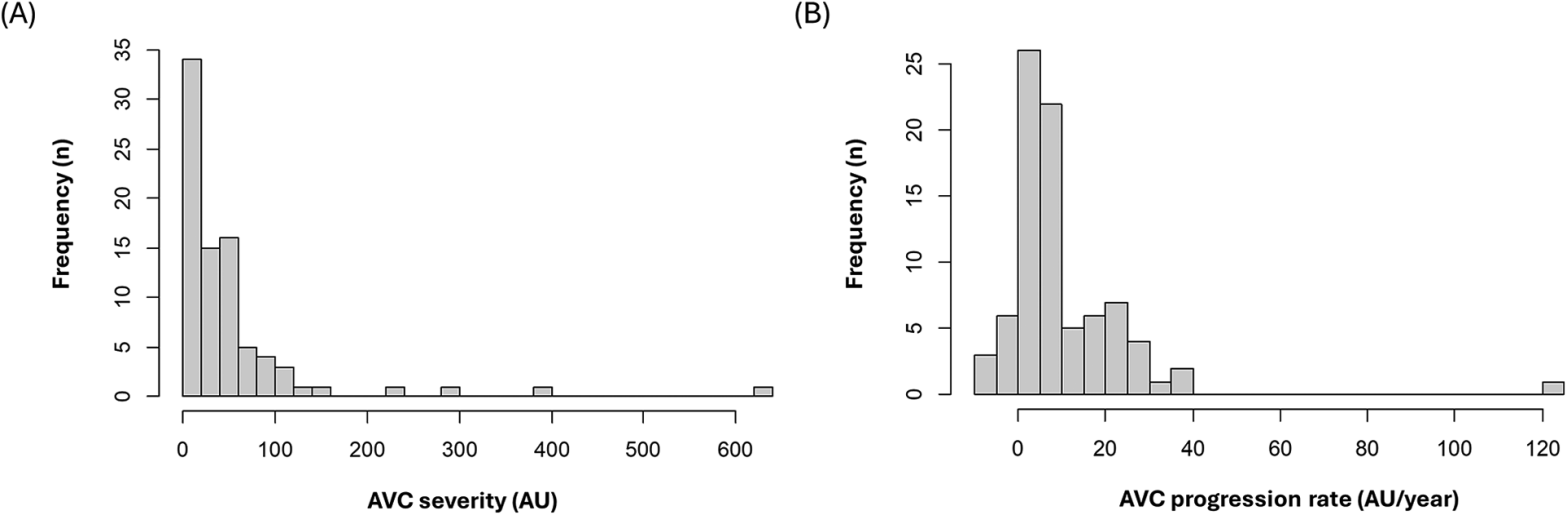
Distribution of **(A)** baseline aortic valve calcification scores **(B)** annualized progression rate of aortic valve calcification scores. AVC, aortic valve calcification; AU, Agatston units.

From the KOICA registry, we selected 6,641 individuals who underwent examination at Seoul St. Mary's Hospital. After excluding two individuals with missing data and two with bicuspid aortic valves, 736 participants with at least two CT scans during the study period (April 2009–July 2016) were included in the final study (Figure 1). The median duration between the initial and final CT was 36.4 [interquartile range (IQR), 23.5–50.3] months, and a total of 1,695 CT scans were considered for analysis. Baseline CAVD was present in 83 individuals (11.2%) on the initial CT scan, and new-onset CAVD developed in 52 participants (7.1%) on subsequent scans. In participants with baseline CAVD, the median AVC score was 30.4 (IQR, 10.6–57.2) Agatston units (AU) on the initial CT (Figure 2A) and increased to 49.4 (IQR, 24.9–92.1) AU on the final CT, with an annualized progression rate of 6.0 (IQR, 2.1–16.8) AU/year (Figure 2B).

The baseline characteristics of the study population are presented in Table 1. The median age of the participants was 57 years, and 80.6% were male, with a low proportion of comorbidities overall. Compared with individuals without CAVD, those with baseline or newly developed CAVD were older, were more likely to have hypertension and higher systolic blood pressure (SBP), and had lower eGFR and higher Framingham Risk Scores (21). On echocardiography, the participants with baseline or newly developed CAVD had a higher proportion of aortic regurgitation, higher left ventricular outflow tract velocities, higher left ventricular mass index (LVMI), and worse diastolic function profiles. Two individuals in the baseline AVC group had mild AS.

**Table 1:** Baseline characteristics of the study population stratified according to the presence of calcified aortic valve disease at baseline and follow-up computed tomography.

	No CAVD	Newly developed CAVD	Baseline CAVD	*p*-value
	(*n* = 601)	(*n* = 52)	(*n* = 83)	
Demographic, clinical, and laboratory characteristics				
Age (years)	53.7 ± 7.9	57.7 ± 7.0	62.6 ± 8.2	<0.001
Sex				0.50
Male (%)	485 (80.7)	39 (75.0)	69 (83.1)	
Female (%)	116 (19.3)	13 (25.0)	14 (16.9)	
BMI (kg/m^2^)	24.7 ± 3.3	24.9 ± 2.8	25.5 ± 2.6	0.08
History of smoking				0.16
Non-smoker	353 (58.7)	36 (69.2)	57 (68.7)	
Ex-smoker	133 (22.1)	8 (15.4)	10 (12.0)	
Current smoker	115 (19.1)	8 (15.4)	16 (19.3)	
Hypertension (%)	169 (28.3)	18 (35.3)	39 (47.0)	<0.01
Diabetes (%)	76 (12.6)	5 (9.6)	18 (21.7)	0.06
Dyslipidemia (%)	104 (17.3)	9 (17.3)	19 (22.9)	0.46
Atrial fibrillation (%)	4 (0.8)	1 (2.1)	1 (1.3)	0.57
Stroke (%)	6 (1.0)	1 (1.9)	2 (2.4)	0.49
CKD (%)	1 (0.2)	1 (1.9)	0 (0.0)	0.06
SBP (mmHg)	123.7 ± 13.0	128.0 ± 13.3	127.1 ± 11.3	0.01
DBP (mmHg)	75.4 ± 9.9	74.9 ± 7.9	74.7 ± 7.8	0.76
WBC (10^9^/L)	5.9 ± 1.7	5.6 ± 1.7	5.9 ± 1.5	0.39
Hemoglobin (mg/dl)	15.0 ± 1.4	14.9 ± 1.1	14.8 ± 1.4	0.41
Platelet (10^9^/L)	232.0 ± 50.3	229.8 ± 49.6	231.6 ± 44.5	0.95
HbA1C (%)	5.8 ± 0.7	5.8 ± 0.7	5.9 ± 0.7	0.15
HDL-C (mg/dl)	49.7 ± 11.3	50.1 ± 10.2	49.4 ± 11.8	0.95
LDL-C (mg/dl)	120.9 ± 32.7	119.8 ± 31.7	121.0 ± 31.6	0.97
Triglycerides (mg/dl)	128.5 ± 80.9	122.6 ± 72.2	133.4 ± 69.7	0.75
Calcium (mg/dl)	9.2 ± 0.4	9.2 ± 0.3	9.2 ± 0.3	0.98
Phosphate (mg/dl)	3.4 ± 0.5	3.6 ± 0.4	3.5 ± 0.5	0.12
eGFR (ml/min/1.73 m^2^)	84.3 ± 10.4	84.2 ± 8.4	80.0 ± 8.7	<0.01
hs-CRP (mg/dl)	0.15 ± 0.34	0.20 ± 0.52	0.17 ± 0.31	0.72
Framingham risk score	11.3 ± 3.5	12.6 ± 3.0	13.6 ± 3.0	<0.001
Echocardiographic characteristics				
AR severity				<0.001
None/trivial (%)	570 (94.8)	46 (88.5)	68 (81.9)	
Mild (%)	31 (5.2)	6 (11.5)	15 (18.1)	
AS severity				<0.001
None (%)	601 (100.0)	52 (100.0)	81 (97.6)	
Mild (%)	0 (0.0)	0 (0.0)	2 (2.4)	
LVOT peak velocity (m/s)	0.94 ± 0.15	0.97 ± 0.19	0.99 ± 0.12	0.03
LVOT VTI (cm)	20.2 ± 3.4	20.9 ± 4.5	21.3 ± 2.9	0.04
LVOT diameter (cm)	2.2 ± 0.2	2.2 ± 0.2	2.2 ± 0.2	0.47
LVEF (%)	65.8 ± 5.4	67.1 ± 4.4	65.9 ± 5.1	0.24
LVEDV (ml)	79.9 ± 19.7	82.8 ± 18.6	79.7 ± 20.9	0.61
LVMI (kg/m^2^)	85.1 ± 18.0	93.8 ± 25.7	92.9 ± 18.2	<0.001
LAVI (ml/m^2^)	24.7 ± 7.4	28.4 ± 9.6	25.8 ± 7.2	0.01
Septal E/e'	8.7 ± 2.3	9.7 ± 2.5	10.2 ± 2.7	<0.001
TR peak velocity (m/s)	2.16 ± 0.25	2.22 ± 0.22	2.25 ± 0.26	0.04

CAVD, calcified aortic valve disease; BMI, body-mass index; CKD, chronic kidney disease; SBP, systolic blood pressure; DBP, diastolic blood pressure; HR, heart rate; WBC, white blood cell; HbA1c, glycated hemoglobin; HDL-C, high-density lipoprotein cholesterol; LDL-C, low-density lipoprotein cholesterol; eGFR, estimated glomerular filtration rate; hs-CRP, high-sensitivity C-reactive protein; AR, aortic regurgitation; AS, aortic stenosis; LVOT, left ventricular outflow tract; VTI, velocity-time integral; LVEF, left ventricular ejection fraction; LVEDV, left ventricular end diastolic volume; LVMI, left ventricular mass index; LAVI, left atrial volume index.

### 3.2 Association of serum phosphate with the probability of calcified aortic valve disease

On univariable logistic regression, there was no clear association between serum phosphate and baseline CAVD, but higher serum phosphate levels were associated with a significantly higher probability of newly developed CAVD (odds ratio [OR] per 1 mg/dl increase, 1.06; 95% confidence interval [CI], 1.01–1.10; *p* = 0.02) (Table 2). This association remained after adjustment for age, sex, hypertension, diabetes, dyslipidemia, and eGFR (model 1a: OR, 1.06; 95% CI, 1.02–1.11; *p* = 0.01); for age, sex, SBP, glycated hemoglobin, low-density lipoprotein cholesterol (LDL-C), and eGFR (model 1b: OR, 1.05; 95% CI, 1.01–1.10; *p* = 0.02); or for the Framingham risk score (21), glycated hemoglobin, and eGFR (model 2: OR, 1.05; 95% CI, 1.01–1.10; *p* = 0.02).

**Table 2:** Association of serum phosphate with the probability of baseline and newly developed calcified aortic valve disease.

	Baseline CAVD		Newly developed CAVD^a^	
	OR (95% CI)	*p*-value	OR (95% CI)	*p*-value
Unadjusted	1.01 (0.96–1.06)	0.66	1.06 (1.01–1.10)	0.02
Model 1a	1.03 (0.98–1.08)	0.29	1.06 (1.02–1.11)	0.01
Model 1b	1.02 (0.97–1.07)	0.43	1.05 (1.01–1.10)	0.02
Model 2	1.00 (0.96–1.05)	0.90	1.05 (1.01–1.10)	0.02
Model 3	1.02 (0.97–1.07)	0.41	1.06 (1.02–1.10)	0.01

^a^ Additionally adjusted for interscan duration in all analyses. Model 1a: adjusted for age, sex, hypertension, diabetes, dyslipidemia, and estimated glomerular filtration rate. Model 1b: adjusted for age, sex, systolic blood pressure, glycated hemoglobin, low-density lipoprotein cholesterol, and estimated glomerular filtration rate. Model 2: adjusted for Framingham risk score, glycated hemoglobin, and estimated glomerular filtration rate. Model 3: adjusted for age, sex, body-mass index, hypertension, low-density lipoprotein cholesterol, and estimated glomerular filtration rate in baseline aortic valve calcification, and for age, systolic blood pressure, left ventricular mass index, and estimated glomerular filtration rate in new-onset aortic valve calcification. CAVD, calcified aortic valve disease; OR, odds ratio.

**Figure 3:**
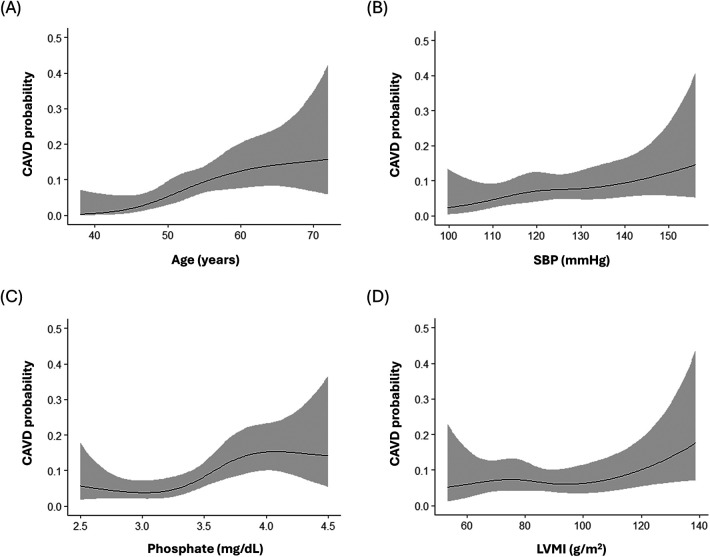
Relationship between the probability of newly developed calcified aortic valve disease and **(A)** age **(B)** systolic blood pressure **(C)** serum phosphate **(D)** left ventricular mass index. SBP, systolic blood pressure; LVMI, left ventricular mass index.

Predictors of baseline and newly developed CAVD other than the traditional atherosclerosis risk factors were identified and adjusted for using a stepwise model (model 3 in Tables 2, 3). Older age (OR per 10 years, 1.15; 95% CI, 1.12–1.19; *p* < 0.001), higher body mass index (BMI) (OR per 5 kg/m^2^, 1.05; 95% CI, 1.02–1.10; *p* < 0.01), history of hypertension (OR, 1.05; 95% CI, 1.00–1.11; *p* = 0.04), and higher LDL-C levels (OR per 10 mg/dl, 1.01; 95% CI, 1.00–1.02; *p* = 0.02) were associated with baseline CAVD; however, serum phosphate levels were not (OR per 1 mg/dl, 1.02; 95% CI, 0.93–1.07; *p* = 0.41). In contrast, higher serum phosphate (OR per 1 mg/dl, 1.06; 95% CI, 1.02–1.10; *p* = 0.01) was a significant predictor for the development of new CAVD, as were older age (OR per 10 years, 1.05; 95% CI, 1.02–1.08; *p* < 0.01), higher SBP (OR per 10 mmHg, 1.04; 95% CI, 1.01–1.07; *p* < 0.01), and higher LVMI (OR per 10 g/m^2^, 1.02; 95% CI, 1.00–1.03; *p* = 0.03). Analysis using restricted cubic splines also showed a positive correlation between age, SBP, serum phosphate, and LVMI with the probability of newly developed CAVD (Figure 3). The increase in the probability of newly developed CAVD with increasing serum phosphate was most prominent between 3.0–4.0 mg/dl and plateaued past this range. The optimal cutoff points for predicting newly developed CAVD were age >51 years, SBP >128 mmHg, serum phosphate >3.6 mg/dl, and LVMI >99 g/m^2^ (Supplementary Material Figure S1), and the C-index for the regression models was 0.79 for baseline CAVD and 0.81 for newly developed CAVD (Supplementary Material Figure S2).

**Table 3:** Factors associated with baseline and newly developed calcified aortic valve disease.

	Baseline CAVD^a^		Newly developed CAVD^b^	
Characteristics	OR (95% CI)	*p*-value	OR (95% CI)	*p*-value
Age (per 10 years)	1.15 (1.12–1.19)	<0.001	1.05 (1.02–1.08)	<0.01
Sex		0.70		0.76
Male	1.01 (0.96–1.07)		1.01 (0.95–1.07)	
Female	Referent		Referent	
BMI (per 5 kg/m^2^)	1.05 (1.02–1.10)	<0.01		
Hypertension	1.05 (1.00–1.11)	0.04		
SBP (per 10 mmHg)			1.04 (1.01–1.07)	<0.01
LDL-C (per 10 mg/dl)	1.01 (1.00–1.02)	0.02		
Phosphate (per 1 mg/dl)	1.02 (0.97–1.07)	0.41	1.06 (1.02–1.10)	0.01
eGFR (per 10 ml/min/1.73 m^2^)	1.02 (0.99–1.04)	0.15	1.02 (1.00–1.05)	0.10
LVMI (per 10 g/m^2^)			1.02 (1.00–1.03)	0.03
Interscan duration (per 1 year)			1.03 (1.02–1.05)	<0.01

^a^ Adjusted for age, sex, body-mass index, hypertension, low-density lipoprotein cholesterol, phosphate, and estimated glomerular filtration rate.

^b^ Adjusted for age, sex, systolic blood pressure, left ventricular mass index, phosphate, and estimated glomerular filtration rate, and interscan duration. OR, odds ratio; CI, confidence interval; AVC, aortic valve calcification; BMI, body-mass index; SBP, systolic blood pressure; LDL-C, low-density lipoprotein cholesterol; eGFR, estimated glomerular filtration rate; LVMI, left ventricular mass index.

### 3.3 Association of serum phosphate with the progression rate of calcified aortic valve disease

In individuals with baseline CAVD, the initial AVC score, serum phosphate, left ventricular end-diastolic volume, and LVMI were associated with a higher annualized AVC score progression rate on univariable linear regression (Table 4). After multivariable adjustment, higher serum phosphate levels (regression coefficient per 1 mg/dl, 15.80; 95% CI, 5.65–25.96; *p* < 0.01), as well as the initial AVC score (regression coefficient per 1 AU, 0.29; 95% CI, 0.26–0.32; *p* < 0.001), male sex (regression coefficient, 15.95; 95% CI, 4.22–27.67, *p* = 0.01), and LVMI (regression coefficient per 10 g/m^2^, 2.31; 95% CI, 0.16–4.45; *p* = 0.04) emerged as factors significantly associated with a higher annualized AVC score progression rate.

**Table 4:** Linear regression for the annualized progression rate of the aortic valve calcification score in the individuals with baseline calcified aortic valve disease.

Risk factors	Unadjusted	*p*-value	Multivariable adjusted^a^	*p*-value
	Regression coefficient (95% CI)		Regression coefficient (95% CI)	
Initial AVC score (per 1 AU)	0.28 (0.25–0.31)	<0.001	0.29 (0.26–0.32)	<0.001
Age (per 10 years)	−6.84 (−17.16–3.49)	0.19	−4.64 (−11.29–2.02)	0.17
Sex		0.25		0.01
Male	13.20 (−9.63–36.04)		15.95 (4.22–27.67)	
Female	referent		referent	
BMI (per 5 kg/m^2^)	6.02 (−11.08–23.13)	0.49		
Smoking	14.60 (−3.49–32.69)	0.11	8.09 (−2.31–18.49)	0.14
Diabetes	−9.10 (−29.91–11.71)	0.39		
Hypertension	−0.25 (−17.41–16.91)	0.99		
Dyslipidemia	5.78 (−16.03–27.59)	0.60		
SBP (per 10 mmHg)	0.82 (−6.92–8.57)	0.83		
DBP (per 10 mmHg)	5.45 (−5.72–16.61)	0.34		
WBC count (per 10^9^/L)	4.92 (−0.67–10.51)	0.08		
Hemoglobin (per 1 mg/dl)	4.18 (−2.14–10.49)	0.19		
HbA1c (per 1%)	−3.66 (−17.30–9.98)	0.59		
LDL-C (per 10 mg/dl)	0.45 (−2.35–3.26)	0.75		
Triglycerides (per 10 mg/dl)	0.10 (−1.18–1.38)	0.88		
eGFR (per 10 ml/min/1.73m^2^)	3.96 (−5.99–13.91)	0.43	0.51 (−4.65–5.67)	0.84
Calcium (per 1 mg/dl)	11.64 (−13.68–36.97)	0.36	−1.03 (−14.22–12.16)	0.88
Phosphate (per 1 mg/dl)	9.93 (1.18–18.69)	0.03	15.80 (5.65–25.96)	<0.01
hs-CRP (per 1 mg/dl)	−3.70 (−26.80–19.40)	0.75		
LVEF (per 1%)	0.52 (−1.35–2.38)	0.58		
LVEDV (per 10 ml)	0.45 (0.02–0.88)	0.04		
LVMI (per 10 g/m^2^)	4.58 (0.34–8.81)	0.03	2.31 (0.16–4.45)	0.04
AR severity		0.16		
None/trivial	referent			
Mild	10.07 (−3.94–24.08)			
LVOT peak velocity (per 10 cm/s)	−4.51 (−12.38–30.13)	0.23		
LVOT VTI (per 1 cm)	−1.91 (−5.09–1.27)	0.24		
Septal E/e’ (per 1)	0.16 (−6.09–1.01)	0.16		
TR peak velocity (per 1 m/s)	28.25 (−17.85–74.34)	0.22		

^a^ Adjusted for initial aortic valve calcification severity, age, sex, history of smoking, estimated glomerular filtration rate, calcium, phosphate, and left ventricular mass index. CI, confidence interval; AVC, aortic valve calcification; AU, Agatston unit; BMI, body-mass index; SBP, systolic blood pressure; DBP, diastolic blood pressure; WBC, white blood cell; HbA1c, glycated hemoglobin; LDL-C, low-density lipoprotein cholesterol; eGFR, estimated glomerular filtration rate; hs-CRP, high-sensitivity C-reactive protein; LVEF, left ventricular ejection fraction; LVEDV, left ventricular end diastolic volume; LVMI, left ventricular mass index; AR, aortic regurgitation, LVOT, left ventricular outflow tract; VTI, velocity-time integral.

**Figure 4:**
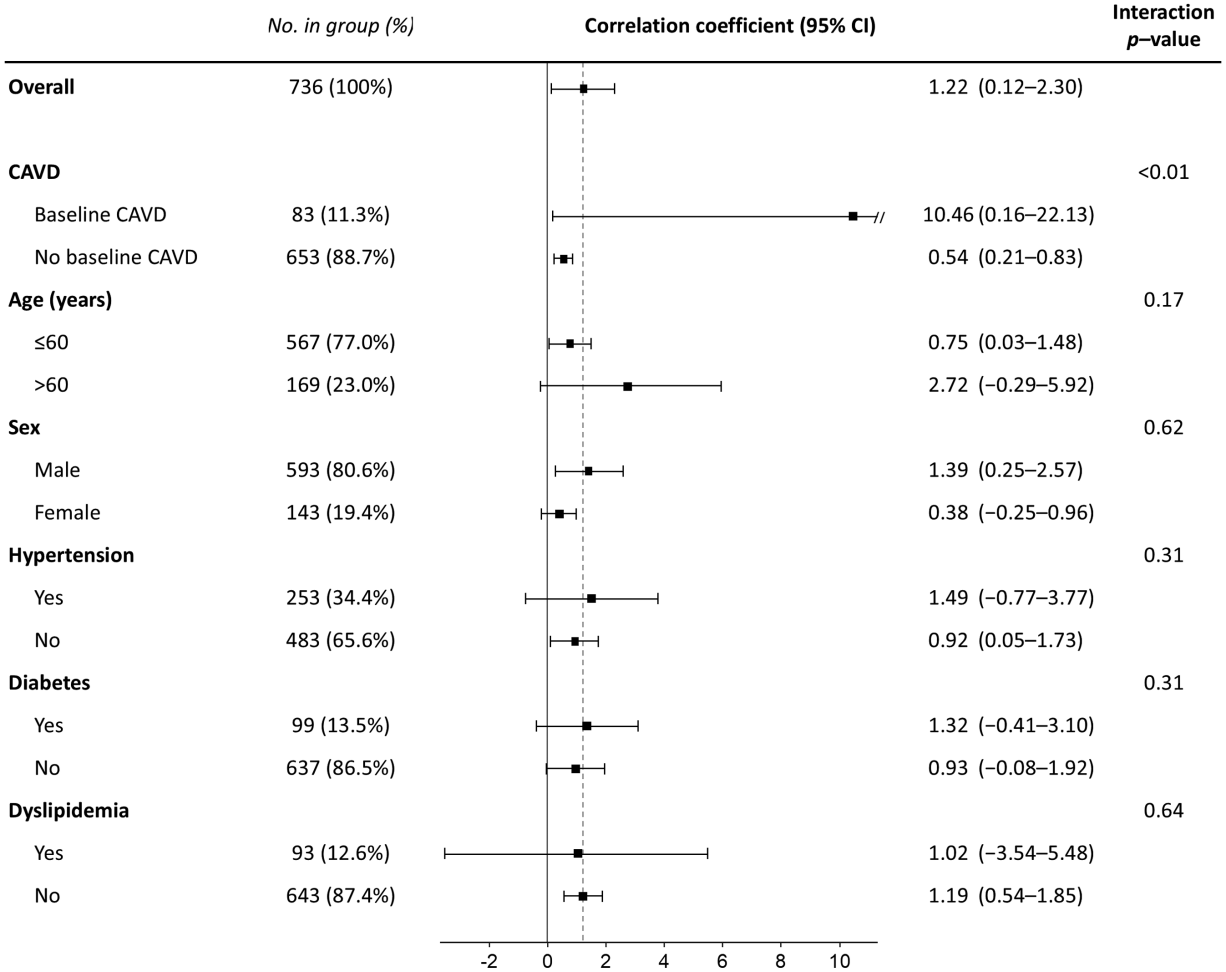
Subgroup analysis for the effect of serum phosphate on the annualized progression rate of the aortic valve calcification score. CI, confidence interval; CAVD, calcified aortic valve disease.

When the association between CAVD progression and serum phosphate levels was analyzed in the entire group including both baseline and newly developed CAVD using a linear mixed-effects model*,* higher serum phosphate was again found to be independently associated with a higher annualized AVC score progression rate (regression coefficient per 1 mg/dl, 1.03; 95% CI, 0.25–1.83; *p* = 0.01) (Table 5). The only other risk factor associated with CAVD progression in this analysis was the initial AVC score (regression coefficient per 1 AU, 0.26; 95% CI, 0.25–0.27; *p* < 0.001). In subgroup analysis, higher phosphate was associated with more rapid progression in both baseline and newly developed CAVD, with a larger effect in those with baseline CAVD (regression coefficient per 1 mg/dl, 10.46; 95% CI, 0.16–22.13; *p* = 0.05) compared with those with newly developed CAVD (regression coefficient per 1 mg/dl, 0.54; 95% CI, 0.21–0.83; *p* < 0.01) (Figure 4). However, the effect of phosphate on CAVD progression was consistent across other subgroups. Finally, as a sensitivity analysis, multivariable linear mixed-effects regression was repeated using log-transformed AVC scores, and serum phosphate remained significantly associated with a higher rate of CAVD progression (*p* < 0.001) (Supplementary Table S1).

**Table 5:** Multivariable linear mixed-effects model for identification of risk factors with the annualized progression rate of the aortic valve calcification score in the entire population.

Risk factors	Regression coefficient (95% CI)	*p*-value
Initial AVC score (per 1 AU)	0.28 (0.25–0.27)	<0.001
Age (per 10 years)	−0.42 (−1.22–0.30)	0.26
Sex		0.22
Male	0.84 (−0.55–2.29)	
Female	referent	
BMI (per 5 kg/m^2^)	−0.32 (−1.20–0.47)	0.45
Smoking	0.53 (−0.06–1.16)	0.10
SBP (per 10 mmHg)	−0.10 (−0.48–0.27)	0.62
HbA1c (per 1%)	−0.53 (−1.24–0.22)	0.15
LDL-C (per 10 mg/dl)	−0.01 (−0.16–0.13)	0.86
eGFR (per 10 ml/min/1.73 m^2^)	0.04 (−0.48–0.60)	0.88
Calcium (per 1 mg/dl)	0.12 (−1.35–1.83)	0.88
Phosphate (per 1 mg/dl)	1.22 (0.12–2.30)	0.02
LVMI (per 10 g/m^2^)	0.15 (−0.11–0.39)	0.27

CI, confidence interval; AVC, aortic valve calcification; AU, Agatston unit; BMI, body-mass index; SBP, systolic blood pressure; HR, heart rate; HbA1c, glycated hemoglobin; LDL-C, low-density lipoprotein cholesterol; eGFR, estimated glomerular filtration rate; LVMI, left ventricular mass index.

### 3.4 Association of serum phosphate levels with echocardiographic parameters

The association between serum phosphate levels and hemodynamic parameters measured using echocardiography are shown in Supplementary Table S2. Depending on the regression model used for adjustment, elevated phosphate had a borderline association with higher E/e’. However, serum phosphate levels did not show a clear relationship with measurements of left ventricular size and function, left ventricular outflow tract velocities, or the probability of aortic regurgitation.

## 4 Discussion

In this retrospective cohort study, we assessed CAVD development using cardiac CT and echocardiography in asymptomatic individuals undergoing self-referred health examination. Higher serum phosphate was associated with the development of new CAVD and a higher annualized AVC score progression rate. We also found differences in factors related to baseline CAVD and the AVC progression rate; the former was most strongly associated with age, BMI, history of hypertension, and LDL-C levels, whereas the latter was associated with the initial AVC score, male sex, serum phosphate levels, and LVMI. Meanwhile, the predictors of new-onset CAVD were intermediate between the two—age, SBP, phosphate, and LVMI.

Previous large cohort studies found that traditional atherosclerotic risk factors, such as older age, higher BMI, smoking, hypertension, and dyslipidemia, are associated with the development of CAVD (8, 22). However, despite the notable overlap, atherosclerosis alone does not fully explain the pathophysiologic process of CAVD. In the Multi-Ethnic Study of Atherosclerosis (MESA), the prime predictor of CAVD progression was the baseline AVC score *per se*, supporting the concept of a self-perpetuating cycle in which calcium begets calcium (8, 9). Our findings are in accord with previous studies in identifying the association of atherosclerotic risk factors with baseline CAVD, and in identifying the baseline AVC score as the most important factor in CAVD progression. The incidence rate of newly developed CAVD (2.3%/year) in the present study was comparable to that reported in MESA (1.7%/year) (8). The identification of male sex and LVMI as a factor in accelerated AVC score progression is also in accord with previous reports (8, 23–25). In addition, we found that higher serum phosphate levels within the normal range were associated with the development of new CAVD and a higher annualized AVC score progression rate, which to the best of our knowledge has not been reported previously.

Since calcification depends on the deposition of calcium-phosphate crystals *in vivo*, phosphate plays an essential role in both physiologic and ectopic vascular calcification (26). The role of elevated phosphate in promoting cardiovascular calcification and as a risk factor for cardiovascular events is well established in patients with chronic kidney disease (27). In addition, a number of previous studies have investigated the relationship between phosphate and CAVD in patients without renal dysfunction. In the Cardiovascular Health Study, higher phosphate levels within the normal range were associated with the presence of CAVD detected on echocardiography (13). Analysis of the Multi-Ethnic Study of Atherosclerosis (MESA) cohort also found that higher serum phosphate was associated with prevalent AVC on CT (14). Although serum phosphate levels are tightly controlled in a relatively narrow range, they are also subject to variation, notably with age (28), and thus the association between the onset of AVC and phosphate may be weakened by time. We speculate that the smaller number of individuals in our study may have resulted in the causative association between AVC and phosphate being lost at the time of baseline examination, while it may have been retained in the MESA study. In the same cohort however, phosphate levels were not associated with the progression of AVC severity (29), and similar results were found in other recent studies (30, 31). Our results are consistent with previous reports in identifying higher phosphate levels as a risk factor in CAVD development, but is also in contrast with these studies in the association of phosphate with accelerated CAVD progression.

One explanation for this apparent discrepancy may be found in the lower baseline AVC scores in our study population (median AVC score 30.4 [IQR 10.6–57.2] vs. 56 [IQR 19–137] in the MESA cohort (8). In the pathogenesis of CAVD, after initial endothelial injury and lipid infiltration, differentiation of aortic valvular interstitial cells into a osteoblast-like phenotype next takes place, a process which elevated phosphate is known to promote (7, 26, 32–36). In contrast, signaling via transforming growth factor β1 and the Wnt/β-catenin pathway in response to mechanical stress is known to be the dominant factor driving calcification in the later stages after osteogenic differentiation has occurred (6, 37–39), which is supported by the correlation between hemodynamic severity and disease progression found in established AS (40–42). There is very limited data on the AVC scores for the prediction of mild AS; however, the presence of mild calcific AS has been found even at AVC scores as low as 100 (43, 44). Thus we may hypothesize that a non-negligible proportion of the MESA cohort may have had more advanced CAVD and elevated transaortic valve pressures, which could have weakened the influence of phosphate on disease progression. However, no significant relationship between CAVD progression and hemodynamic parameters measured on echocardiography was found in our analysis. Thus, we suggest that our population may be representative of CAVD in the earlier stages of development where osteoblast differentiation is the main pathophysiologic mechanism, and which may possibly be affected by serum phosphate levels. Alternatively, there is a possibility that our results are specific to the East Asian population. Previous studies have found that there are ethnic differences in bone-mineral metabolism (45, 46), which may be an explanation for the different results found in our analysis.

It is not clear whether the association between phosphate levels and CAVD progression found in our study is directly causative in nature, or whether it reflects another underlying process in mineral metabolism. However, serum phosphate is widely measured in clinical practice, and elevated phosphate levels in patients with CAVD may indicate a higher risk of accelerated CAVD progression, which may potentially benefit from therapeutic interventions targeting various pathways in mineral metabolism (12). Further studies are needed to investigate whether the regulation of phosphate metabolism can slow the progression of early CAVD to AS.

Despite the findings of our study, it had several limitations. First, as previously mentioned, the severity of CAVD in our study population was generally low, and due to the small number of participants with higher AVC scores, we could not perform subgroup analysis to investigate the effect of phosphate levels according to the severity of CAVD or determine cut-off points. Second, although various hemodynamic parameters were included in our analysis, the effect of peak transaortic velocities on CAVD could not be investigated because our study population did not include significant AS and only a small proportion of the population had continuous-wave Doppler transaortic velocity measurements. However, we analyzed the effect of left ventricular outflow tract velocities and diastolic profiles and found no association between these parameters and CAVD progression. Although these factors have been linked with accelerated AS progression in previous studies (41, 42), our negative results suggest that hemodynamic effects may not be significant at least in the early stages of CAVD. Third, we only adjusted for the effect of laboratory and echocardiographic variables measured at baseline, which may have changed during the follow-up period. Lastly, owing to the retrospective nature of the study, there was heterogeneity in patient characteristics and duration to follow-up examination. Although we used multivariable regression to adjust for confounders, we cannot exclude the possibility of remaining bias.

## 5 Conclusion

In a cohort of asymptomatic individuals undergoing health examination, atherosclerotic risk factors were strongly associated with baseline CAVD. Upper normal phosphate levels were associated with the development of new CAVD and the accelerated progression of existing CAVD. The effect of phosphate on CAVD progression may be due to the lower baseline AVC scores in our study population, as the dominant pathophysiologic mechanism driving calcification may be different across the stages of CAVD development. Further studies are needed to confirm these findings, and to investigate whether therapeutic interventions targeting phosphate metabolism can slow the progression of early CAVD to AS.

## Data Availability

The datasets presented in this article are not readily available due to the conditions of the IRB approval, as the dataset contains personal data. Requests to access the datasets should be directed to the corresponding author.
