# Supplementary material for: Higher serum phosphate within the normal range is associated with the development of calcified aortic valve disease
Source: Front Cardiovasc Med. 2024 Sep 26;11:1450757. doi: 10.3389/fcvm.2024.1450757 (PMC11467965; doi:10.3389/fcvm.2024.1450757)
Supplement: Supplementary file 1 [file Datasheet1.pdf]

Supplementary Material

Statistical analysis

The relationship between serum phosphate and calcified aortic valve disease (CAVD) development was explored in a number of ways. First, the association of phosphate with both baseline and newly developed CAVD was analyzed using multivariable logistic regression. In Model 1, we adjusted for known risk factors of CAVD: age, sex, hypertension, diabetes, and dyslipidemia, as well as estimated glomerular filtration rate (eGFR) (Model 1a); or alternately with systolic blood pressure (SBP), glycated hemoglobin (HbA1c), and low-density lipoprotein cholesterol (LDL-C) in place of hypertension, diabetes, and dyslipidemia, to account for the possible attenuating effect of medications (Model 1b). In Model 2, to avoid overfitting due to the small number of individuals who developed new CAVD, adjustment was made for the Framingham Risk Score,[1] as well as HbA1c and eGFR. In order to identify and adjust for other variables beside the traditional atherosclerosis risk factors, in Model 3 we entered each baseline variable with $p>0.20$ in univariable analysis into the multivariable model, and a backward stepwise elimination process was used with the stepwise Akaike information criterion method while retaining phosphate and eGFR, as well as age and sex. In each model, the probability of newly developed CAVD was additionally adjusted for the interscan duration. The optimal cutoff value for each significant variable was determined by points on the receiver operating characteristic curve maximizing the Youden index. Restricted cubic splines were also constructed using logistic regression with the variables determined in Model 3 to visualize the relationship between serum phosphate and other significant predictors with the probability of newly developed CAVD. The spline functions were defined such that each function is piecewise cubic between the knots and linear beyond the boundary knots, with knots placed at the 5th, 35th, 65th, and 95th percentiles. The smoothness of the splines was ensured by imposing the conditions that that the piecewise cubic polynomial function, and the first and second order derivatives of the function, are continuous at each knot. Confidence intervals were obtained using the variance-covariance matrix of model coefficients to compute the standard errors of predictions.

Second, the association of serum phosphate levels with the progression of CAVD was analyzed using multivariable linear regression on the annualized progression rate of the aortic valve calcification (AVC) score using the first and last CT scans in those with baseline AVC. Previous studies have found that no factors other than baseline AVC severity were definitely associated with AVC progression.[2] Since we had no a priori expectations on which factors would contribute to a higher rate of AVC progression, all variables with $p<0.2$ were entered into multivariable analysis, and a backward elimination process with the Akaike information criterion was used to select the final variables to avoid overfitting. Calcium and eGFR was retained in the model to adjust for the effect of renal function on serum phosphate, as well as age and sex. Collinearity was assessed using the variance inflation factor (VIF), which was <5 for each variable in the final model. Homoskedasticity was also assessed and satisfied using White's test and by plots of the residuals and fitted values.

Third, the association of CAVD progression with serum phosphate levels was analyzed in the entire group, including all available CT scans, using a multivariable linear mixed-effects model with individual intercepts and the repeated effect estimated for time. This approach has been used in previous studies on coronary and cardiac valve calcifications and has the advantage of using all available data without the need to analyze de novo CAVD and progression from baseline CAVD separately.[3] The variables included in the model were the initial AVC score, age, sex, body-mass index (BMI), smoking history, systolic blood pressure, HbA1c, LDL-C, eGFR, calcium, phosphate, and left ventricular mass index (LVMI). The regression equation used for the multivariable linear mixed-effect model is $Y_{ij} = \beta_0 + \beta_1 t_{ij} + \beta_{2i} x_i + \beta_{3i} t_{ij} x_i + b_i + \varepsilon_{ij}$, where the dependent variable Y_{ij} is the jth measurement of the AVC score in the ith subject, b_i the random intercept for subject i, and ε_{ij} the individual error term. The main predictors are years after baseline examination (t_{ij}), demographic or laboratory risk factors (x_i), and the interaction term between the two. The estimated values of the regression coefficient β_{3i} are presented in Table 4. Confidence intervals for the multivariable linear mixed-effects model were calculated using a parametric bootstrap method. Our main approach was to use the AVC score in its original form for ease of interpretation; however we also used log-transformed AVC scores, specifically log(AVC score + 1), due to the skewed distribution of the AVC scores. Subgroup analysis was also performed using the same multivariable linear mixed-effects model to confirm the effect of phosphate

levels on CAVD progression in each subgroup, according to the presence of CAVD at baseline, age above or below 60, sex, and history of hypertension, diabetes, and dyslipidemia. The probability of an interaction effect was estimated using multiplicative interaction terms for each subgroup.

The relationship between serum phosphate levels and echocardiographic parameters was also investigated using multivariable regression models. The probability of aortic regurgitation was analyzed using logistic regression, while parameters with numerical values were analyzed using linear regression. In Model 1, we adjusted for age, sex, hypertension, diabetes, dyslipidemia, and eGFR, while in Model 2, we adjusted for age, sex, SBP, HbA1c, LDL-C, and eGFR.

Statistical analyses were performed using R version 4.3.2 (R Foundation for Statistical Computing, Vienna, Austria), with packages “rms”, “Epi”, “lme4”, and “afex”.

Table S1. Multivariable linear mixed-effects model for identification of risk factors with the annualized progression rate of the aortic valve calcification score in the entire population with log-transformed aortic valve calcification scores

Risk factors	100 × Regression coefficient (95% CI) *	p-value
Log (Initial AVC score + 1) (per 1 AU)	1.65 (0.32-2.98)	0.02
Age (per 10 years)	5.19 (3.11-7.21)	<0.01
Sex		0.68
Male	-0.78 (-4.68-2.69)	
Female	referent	
BMI (per 5kg/m^2)	3.21 (0.83-5.72)	0.01
Smoking	1.71 (0.00-3.48)	0.06
SBP (per 10mmHg)	0.68 (-0.29-1.83)	0.21
HbA1c (per 1%)	-1.24 (-3.24-0.58)	0.21
LDL-C (per 10mg/dL)	-0.34 (-0.79-0.07)	0.12
eGFR (per 10mL/min/1.73m^2)	1.40 (-0.14-3.03)	0.06
Calcium (per 1mg/dL)	0.42 (-3.48-4.73)	0.84
Phosphate (per 1mg/dL)	5.98 (3.13-8.81)	<0.001
LVMI (per 10g/m^2)	-0.03 (-0.11-0.04)	0.38

* Adjusted for all variables simultaneously. Regression coefficients are multiplied by 100 in this table due to the small values of log-transformed aortic valve calcification scores. CI, confidence interval; AVC, aortic valve calcification; AU, Agatston unit; BMI, body-mass index; SBP, systolic blood pressure; HR, heart rate; HbA1c, glycated hemoglobin; LDL-C, low-density lipoprotein cholesterol; eGFR, estimated glomerular filtration rate; LVMI, left ventricular mass index.

Table S2. Association of serum phosphate levels with echocardiographic parameters

Parameter	Model 1*		Model 2†	
	Regression coefficient (95% CI) ‡	p-value	Regression coefficient (95% CI) ‡	p-value
LVEF (per 1%)	-0.46 (-1.29-0.37)	0.28	-0.41 (-1.28-0.46)	0.35
LVEDV (per 1ml)	0.84 (-2.13-3.81)	0.58	0.17 (-2.82-3.16)	0.91
LVMI (per 1g/m^2)	-0.03 (-2.97-2.92)	0.99	-0.86 (-3.79-2.07)	0.57
LVOT peak velocity (per 1cm/s)	0.00 (-0.03-0.03)	0.89	0.00 (-0.03-0.03)	0.87
LVOT VTI (per 1cm)	-0.34 (-1.05-0.37)	0.35	-0.27 (-0.98-0.44)	0.45
E/e’ (per 1)	0.47 (0.11-0.83)	0.01	0.35 (-0.02-0.72)	0.06
TR peak velocity (per 1m/s)	-0.01 (-0.06-0.04)	0.69	0.00 (-0.05-0.05)	0.97
	OR (95% CI)	p-value	OR (95% CI)	p-value
Aortic regurgitation§	1.03 (0.99-1.07)	0.11	1.03 (0.99-1.06)	0.12

* Model 1: adjusted for age, sex, hypertension, diabetes, dyslipidemia, and estimated glomerular filtration rate. † Model 2: adjusted for age, sex, systolic blood pressure, glycated hemoglobin, low-density lipoprotein cholesterol, and estimated glomerular filtration rate. ‡ Per 1mg/dL increase in serum phosphate. § For the presence of mild aortic regurgitation or greater. CI, confidence interval; LVEF, left ventricular ejection fraction; LVEDV, left ventricular end-diastolic volume; LVMI, left ventricular mass index; LVOT, left ventricular outflow tract; VTI, velocity-time integral; TR, tricuspid regurgitation; OR, odds ratio.

Figure S1. Receiver operating characteristic curves of individual risk factors for the prediction of newly developed calcified aortic valve disease

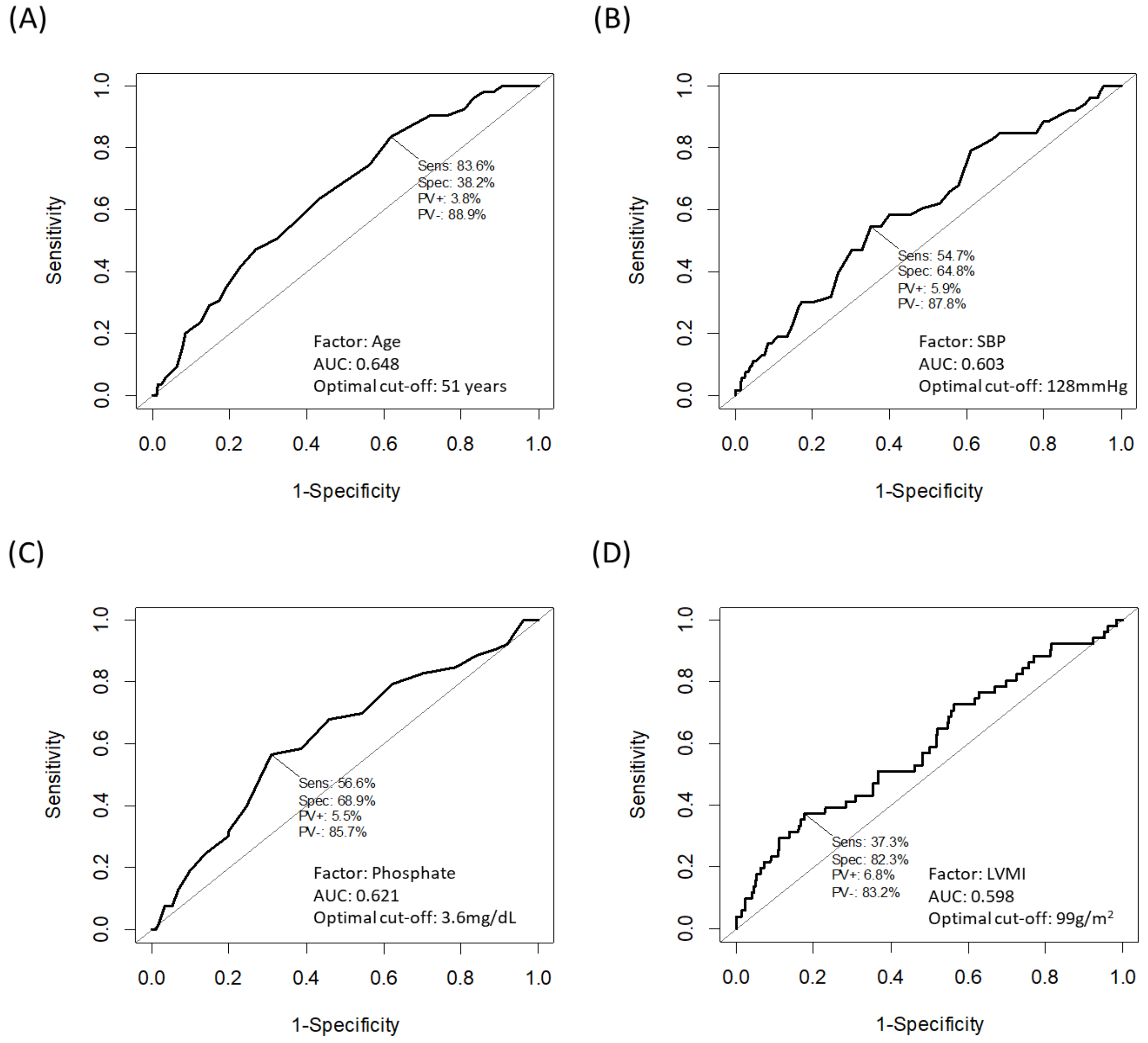

Confidence intervals are calculated using DeLong's method. AUC, area under the curve; SBP, systolic blood pressure; LVMI, left ventricular mass index.

Figure S2. Receiver operating characteristic curves of the logistic regression models for (A) baseline calcified aortic valve disease (B) newly developed calcified aortic valve disease

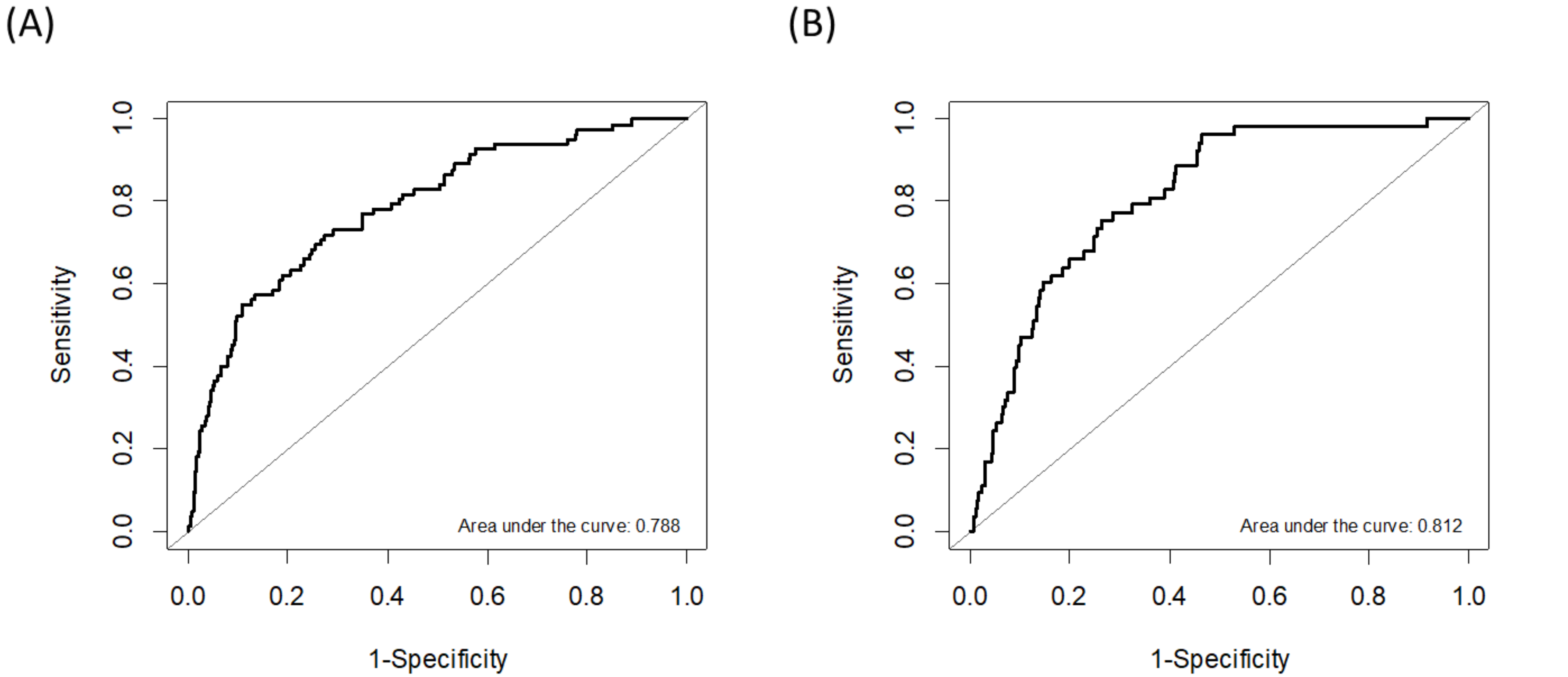

(A) Model includes age, sex, body-mass index, hypertension, low-density lipoprotein cholesterol, phosphate, and estimated glomerular filtration rate. (B) Model includes age, sex, systolic blood pressure, left ventricular mass index, phosphate, and estimated glomerular filtration rate.

References

1. D'Agostino RB, Sr., Vasan RS, Pencina MJ, Wolf PA, Cobain M, Massaro JM, Kannel WB. General cardiovascular risk profile for use in primary care: the Framingham Heart Study. *Circulation*. 2008;117:743-753. doi: 10.1161/CIRCULATIONAHA.107.699579
2. Owens DS, Katz R, Takasu J, Kronmal R, Budoff MJ, O'Brien KD. Incidence and progression of aortic valve calcium in the Multi-ethnic Study of Atherosclerosis (MESA). *Am J Cardiol*. 2010;105:701-708. doi: 10.1016/j.amjcard.2009.10.071
3. Bortnick AE, Xu S, Kim RS, Kestenbaum B, Ix JH, Jenny NS, de Boer IH, Michos ED, Thanassoulis G, Siscovick DS, et al. Biomarkers of mineral metabolism and progression of aortic valve and mitral annular calcification: The Multi-Ethnic Study of Atherosclerosis. *Atherosclerosis*. 2019;285:79-86. doi: 10.1016/j.atherosclerosis.2019.04.215
